# Evaluation of using composite HPV genotyping assay results to monitor human papillomavirus infection burden through simulation

**DOI:** 10.1186/s12879-015-0851-x

**Published:** 2015-03-12

**Authors:** Carol Y Lin

**Affiliations:** Division of STD Prevention, National Center for HIV, Viral Hepatitis, STD, and TB Prevention, Centers for Disease Control and Prevention, 1600 Clifton Road, NE, MS E-63, Atlanta, GA 30333 USA

**Keywords:** HPV prevalence, PCR genotyping assay, Vaccine effectiveness, Sensitivity, Specificity

## Abstract

**Background:**

Researchers often group various HPV types into composite measures based on vaccine subtypes, oncogenic potential, or phylogenetic position. Composite prevalence estimates based on PCR genotyping assay results have been calculated to assess HPV infection burden and to monitor HPV vaccine effectiveness. While prevention and intervention strategies can be made based on these prevalence estimates, the discussion on how well these prevalence estimates measure the true underlying infection burdens is limited.

**Methods:**

A simulation study was conducted to evaluate accuracy of using composite genotyping assay results to monitor HPV infection burden. Data were generated based on mathematical algorithms with prespecified type-specific infection burdens, assay sensitivity, specificity, and correlations between various HPV types. Estimated-to-true prevalence rate ratios and percent reduction of vaccine types were calculated.

**Results:**

When “true” underlying type-specific infection burdens were prespecified as the reported prevalence in U.S. and genotyping assay with sensitivity and specificity (0.95, 0.95) was used, estimated-to-true infection prevalence ratios were 2.35, 2.29, 2.18, and 1.46, for the composite measures with 2 high-risk vaccine, 4 vaccine, 14 high-risk and 37 HPV types, respectively. Estimated-to-true prevalence ratios increased when prespecified “true” underlying infection burdens or assay specificity declined. When prespecified “true” type-specific infections of HPV 6, 11, 16 and 18 were reduced by 50%, the composite prevalence estimate of 4 vaccine types only decreased by 17% which is much lower than 48% reduction in the prespecified “true” composite prevalence.

**Conclusions:**

Composite prevalence estimates calculated based on panels of genotyping assay results generally over-estimate the “true” underlying infection burdens and could under-estimate vaccine effectiveness. Analytical specificity of genotyping assay is as or more important than analytical sensitivity and should be considered in selecting assay to monitor HPV.

## Background

Infection with human papillomaviruses (HPVs) can cause warts and various forms of carcinoma in the cervix, anus, vagina, vulva, head and neck in women and men [1]. More than 120 types of HPV have been identified according to DNA genomes [1,2]. Researchers have classified and grouped these HPV types by their association with a variety of clinical conditions (i.e., cervical cancer, warts), phylogenetic position, and types related to vaccines [1,3] to form composite measures. Polymerase chain reaction (PCR-based) DNA genotyping tests can detect the existence of small amount of virus and have been considered as the “gold standard” to detect infectious organisms [4,5]. Prevalence estimates based on one or a panel of PCR genotyping test results has been used for research purposes in numerous epidemiology/clinical studies to assess the burden of HPV infections [6-10] and vaccine effectiveness [11-13]. While prevention and intervention strategies can be made based on these prevalence estimates, research on how well these composite prevalence estimates measure the true underlying infection burden is limited.

Various factors could affect accuracy of composite prevalence estimates. Depending on the classification, the number of HPV types included in the composite measures could be different. Since HPV type-specific infections share the same risk factors (i.e., sexual lifestyle, age, etc.) and subjects with weaker immune systems are more likely to get infected or stay infected, HPV type-specific infections are likely to be correlated and could result in coinfection with more than one HPV type [14,15]. In another word, the probability of getting infected or detecting any given HPV type is greater among individuals who are currently positive for at least one other HPV type. In addition, the underlying infection prevalence of various HPV types can be different by study population (i.e., different age groups and geographic regions) or change over time [16,17]. Furthermore, more than 20 in-house or commercial HPV genotyping assays (e.g., Linear Array, INNO-LiPA) have been used to detect HPV infections [13,16,18,19]. Analytical sensitivity and specificity of these genotyping assays can vary greatly for various reasons (e.g., primer set, reaction condition, laboratory techniques of personnel, etc.) [18-20] and result in different composite prevalence estimates.

Challenges to evaluate accuracy of prevalence estimates include not knowing the true values of underlying type-specific infection burdens, low feasibility to recruit subjects from various regions with various levels of infection burdens and to test numerous assays for comparisons. To overcome these challenges, we performed a Monte Carlo simulation study to evaluate composite prevalence estimates. The simulation approach allows us to have data with various levels of known prespecified “true” underlying type-specific infection burdens and genotyping test results based on the prespecified assay performance so the prevalence estimates calculated based on type-specific assay results can be compared to the pre-specified “true” underlying infection burden. Monte Carlo simulation approach has been widely used in statistics, physics, finance, economics and engineering to evaluate the impact of various factors on complex systems/processes and identify an optimal system design/process [21-25]. For this study, we are interested in examining accuracy of the composite prevalence estimates based on panels of PCR genotyping assay results and the impacts of various factors.

## Method

### Simulation settings

A simulation study was conducted to evaluate composite prevalence estimates calculated based on panels of PCR genotyping assay results. Data were generated using mathematical algorithms extended from Lin et al. [26] with prespecified values of “true” underlying type-specific prevalence, PCR genotyping assay sensitivity and specificity and correlations among HPV types. The infectious statuses were assigned based on the prespecified type-specific prevalence; and the genotyping test results were obtained based on the type-specific infectious status, genotyping sensitivity and specificity. Each data set included the known “true” type-specific infection statuses and genotyping assay results so the prevalence estimates could be calculated to compare to the prespecified “true” infection burdens. Without loss of generality, the total number of subjects was set to be 4,000 and correlations between HPV types were set to be 0.05 which was the average value of 666 pairwise correlations of 37 HPV genotyping test results in the 2003–2006 National Health and Nutrition Examination Survey (NHANES). For each scenario, 500 data sets were generated. Mean and standard deviation of the “true” and estimated composite prevalence of 500 data sets were calculated. Mathematical algorithms related to the simulation setup are given in the Appendix.

**Figure 1:**
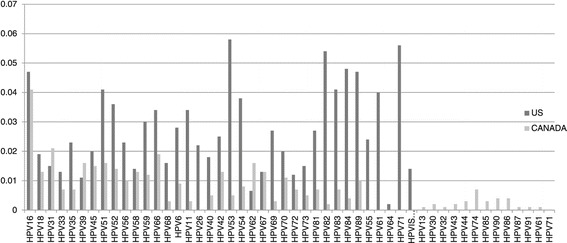
**The reported type-specific prevalence of the U.S., 2003–2006 and the Northwest Territories (NWT), Canada, 2008–2009.**

Two different levels of infection burdens were considered. For the first setting, the “true” underlying type-specific infection burdens were prespecified as the reported 37 different HPV prevalence of females, aged 14–59, 2003–2006, in the U.S. (Figure 1) [7]. For the second setting, the “true” underlying type-specific infection burdens were prespecified as the reported 45 different HPV prevalence of females, aged 14 or older, 2008–2009 in the Northwest Territories (NWT), Canada [9]. The reported type-specific prevalence in the NWT, Canada was generally lower than in the U.S. and the relative rates were also different (Figure 1).

### Definition of true and estimated composite prevalence

Four composite outcome measures are defined as the following: having HPV (37 types in the U.S. and 45 types in Canada), having high-risk HPV (14 types in the U.S. and 22 types in Canada), having vaccine type HPV (HPV 6, 11, 16 and 18), and having high-risk vaccine type HPV (HPV 16, 18). HPV 16 and HPV 18 are generally considered particularly high-risk genotypes and account for approximately 70% of invasive cervical cancers globally [27,28]. Quadrivalent HPV Vaccine (HPV4, Gardasil) protects against HPV 6, 11, 16 and 18. Bivalent HPV (HPV2, Cervarix) protects against HPV 16 and 18.

Prespecified “true” prevalence of each outcome measure is defined as the proportion of subjects with a positive infection status. For each subject, based on the pre-specified “true” type-specific infectious statuses, the “true” composite positive infection status of the four outcome measures is defined as having at least one HPV type-specific infection of the 37 HPV types (45 HPV in Canada), 14 high-risk types (22 high-risk in Canada), 4 vaccine types, and 2 high-risk vaccine types.

Prevalence estimate of each outcome measure is defined as the proportion of subjects with positive composite test results. For each subject, a positive test result of the four outcome measure is defined as having at least one positive of the 37 HPV (45 HPV in Canada), 14 high-risk (22 high-risk in Canada), 4 vaccine, and 2 high-risk vaccine type-specific results. To examine how well the composite prevalence estimate measures the “true” underlying composite prevalence, for each outcome measure, estimated-to-true prevalence ratio was calculated. A ratio greater than 1 means the composite prevalence estimate calculated based on a panel of genotyping assay results over-estimates the “true” underlying composite infection burden. The number of false positives exceeds the number of false negatives. In contrast, a ratio less than 1 means composite prevalence estimate calculated based on a panel of genotyping assay results underestimates the “true” underlying composite infection burden. The number of false negatives exceeds the number of false positives. The larger the ratio from 1, the less accurate the composite prevalence estimate is.

To assess the effects of analytical sensitivity and specificity of genotyping assay on composite prevalence estimates, the initial values of PCR genotyping assay sensitivity and specificity were set to be equal (0.95, 0.95) to reflect the well-performed Linear Array test and then the assay performance was varied. To examine the effect of genotyping assay specificity, assay sensitivity was held unchanged and specificity was reduced from 0.95 to 0.90, 0.85 and 0.80. Similarly, to examine the effect of genotyping assay sensitivity, assay specificity was held unchanged and sensitivity was reduced from 0.95 to 0.90, 0.85 and 0.80.

To examine how well the composite prevalence estimates measured intervention effectiveness, for demonstration purposes, assuming the pre-specified “true” type-specific prevalence of the 4 vaccine types (HPV 6, 11, 16 and 18) were reduced by 50%. Percent reductions of composite prevalence estimates of 4 vaccine types and 2 high-risk vaccine types (HPV 16, 18) were calculated and compare to the reductions in the prespecified “true” underlying composite prevalence.

To examine the effects of levels of underlying type-specific infection burdens and number of HPV types in the composite measure on the composite prevalence estimates, prevalence estimates of the four outcome measures from the U.S. scenarios were compared to those estimates from the NWT, Canada scenarios. The reported type-specific HPV prevalence in the NWT, Canada was generally lower than in the U.S. and the relative rates were also different (Figure 1). In the U.S. scenarios, there are 37 HPV types and 14 high-risk types. In the NWT, Canada, there are 45 HPV types and 22 high-risk types.

To examine the effects of correlations between different HPV types on composite prevalence estimates, the correlations between different HPV types were varied from 0, 0.05, 0.1, 0.2, 0.3 and 0.4. The “true” underlying type-specific infections were prespecified as the reported prevalence in the U.S. and type-specific assay sensitivity and specificity were set to be (0.95, 0.95). “True” composite prevalence, estimated composite prevalence and estimated-to-true prevalence ratios of 37 HPV, 14 high-risk HPV, 4 vaccine and 2 high-risk vaccine types were calculated.

## Results

Table 1(a,b) show the results of estimated composite prevalence when the “true” type-specific infection burdens are prespecified as the reported prevalence in the U.S. When genotyping assay with sensitivity and specificity (0.95, 0.95) is used, composite prevalence estimates based on a panel of genotyping assay results generally overestimate prespecified “true” composite infection burden. As shown in Table 1, the estimated-to-true prevalence rate ratios are 2.35, 2.29, 2.18 and 1.46 for composite measures with 2, 4, 14 and 37 HPV types, respectively.

**Table 1:** **Composite prevalence estimates when the “true” underlying type-specific infection burden is pre-specified as the reported prevalence in the U.S.**

No. of HPV types*	**Time** ^**§**^	**Pre-specified true composite prevalence (SD)**	**Composite prevalence estimate (SD)**	**Est. to true ratio**	**Composite prevalence estimate (SD)**	**Est. to true ratio**	**Composite prevalence estimate (SD)**	**Est. to true ratio**	**Composite prevalence estimate (SD)**	**Est. to true ratio**
**a: Sensitivity ≤ Specificity**										
			**Sen.= 0.95**		**Sen. = 0.90**		**Sen. = 0.85**		**Sen. = 0.80**	
			**Spe.= 0.95**		**Spe. = 0.95**		**Spe. = 0.95**		**Spe. = 0.95**	
2	Baseline	0.064 (0.004)	0.151 (0.006)	2.359	0.148 (0.005)	2.313	0.146 (0.006)	2.281	0.143 (0.005)	2.234
	Reduced	0.032 (0.003)	0.125 (0.005)	3.906	0.123 (0.005)	3.843	0.121 (0.005)	3.781	0.120 (0.005)	3.750
	% red.	50	17.2		16.9		17.1		13.9	
4	Baseline	0.120 (0.005)	0.275 (0.007)	2.292	0.269 (0.007)	2.242	0.264 (0.007)	2.200	0.260 (0.007)	2.167
	Reduced	0.062 (0.004)	0.229 (0.007)	3.694	0.227 (0.006)	3.661	0.224 (0.007)	3.613	0.222 (0.006)	3.581
	% red.	48	16.7		15.6		15.2		14.6	
14	Baseline	0.282 (0.007)	0.616 (0.007)	2.184	0.609 (0.008)	2.160	0.603 (0.008)	2.138	0.596 (0.008)	2.113
	Reduced	0.259 (0.007)	0.604 (0.008)	2.332	0.599 (0.008)	2.313	0.593 (0.008)	2.290	0.587 (0.008)	2.266
	% red.	8.2	1.9		1.6		1.7		1.5	
37	Baseline	0.617 (0.008)	0.904 (0.004)	1.465	0.900 (0.005)	1.459	0.895 (0.005)	1.451	0.891 (0.005)	1.444
	Reduced	0.596 (0.008)	0.899 (0.005)	1.508	0.895 (0.005)	1.502	0.891 (0.005)	1.495	0.887 (0.005)	1.488
	% red.	3.4	0.5		0.6		0.4		0.4	
**b: Sensitivity ≥ Specificity**										
			**Sen. = 0.95**		**Sen. = 0.95**		**Sen. = 0.95**		**Sen. = 0.95**	
			**Spe. = 0.95**		**Spe. = 0.90**		**Spe. = 0.85**		**Spe. = 0.80**	
2	Baseline	0.064 (0.004)	0.151 (0.006)	2.359	0.237 (0.007)	3.703	0.318 (0.007)	4.969	0.394 (0.008)	6.156
	Reduced	0.032 (0.003)	0.125 (0.005)	3.906	0.213 (0.006)	6.656	0.297 (0.007)	9.281	0.374 (0.008)	11.688
	% red.	50	17.2		10.1		6.6		5.1	
4	Baseline	0.120 (0.005)	0.275 (0.007)	2.292	0.409 (0.008)	3.408	0.525 (0.008)	4.375	0.621 (0.008)	5.175
	Reduced	0.062 (0.004)	0.229 (0.007)	3.694	0.374 (0.008)	6.033	0.496 (0.008)	8.000	0.598 (0.008)	9.645
	% red.	48	16.7		8.6		5.5		3.7	
14	Baseline	0.282 (0.007)	0.616 (0.008)	2.184	0.797 (0.006)	2.826	0.895 (0.005)	3.174	0.947 (0.004)	3.358
	Reduced	0.259 (0.006)	0.604 (0.008)	2.332	0.792 (0.006)	3.058	0.892 (0.005)	3.444	0.945 (0.004)	3.649
	% red.	8.2	1.9		0.6		0.3		0.2	
37	Baseline	0.617 (0.008)	0.904 (0.004)	1.465	0.975 (0.002)	1.580	0.993 (0.001)	1.609	0.998 (0.007)	1.515
	Reduced	0.596 (0.008)	0.899 (0.005)	1.508	0.974 (0.003)	1.634	0.993 (0.001)	1.666	0.998 (0.007)	1.674
	% red.	3.4	0.5		0.1		0		0	

*2: high-risk vaccine types (HPV 16, 18); 4: vaccine types (HPV 6, 11, 16, 18); 14: high-risk types (HPV 16, 18, 31, 33, 35, 39, 45, 51, 52, 56, 58, 59, 66, 68); 37: HPV types (HPV 6, 11, 16, 18, 26, 31, 33, 35, 39, 40, 42, 45, 51, 52, 53, 54, 55, 56, 58, 59,61, 62, 64, 66, 67, 68, 69, 70, 71, 72, 73, 81, 82, 83, 84, 89, IS39).

^§^Pre-specified type-specific prevalence at baseline: HPV 6 = 0.028, HPV11 = 0.034, HPV16 = 0.047, HPV18 = 0.019, HPV26 = 0.022, HPV31 = 0.015, HPV33 = 0.013, HPV35 = 0.023, HPV39 = 0.011, HPV40 = 0.018, HPV42 = 0.025, HPV45 = 0.020, HPV51 = 0.041, HPV52 = 0.036, HPV53 = 0.058, HPV54 = 0.038, HPV55 = 0.024, HPV56 = 0.023, HPV58 = 0.014, HPV59 = 0.030, HPV61 = 0.040, HPV62 = 0.065, HPV64 = 0.002, HPV66 = 0.034, HPV67 = 0.013, HPV68 = 0.016, HPV69 = 0.027, HPV70 = 0.020, HPV71 = 0.056, HPV72 = 0.012, HPV73 = 0.015, HPV81 = 0.027, HPV82 = 0.054, HPV83 = 0.041, HPV84 =0.048, HPV89 = 0.047, HPVIS39 = 0.014.

Reduced: vaccine types (HPV 6, 11, 16, 18) are reduced 50%.

When assay specificity is held unchanged and sensitivity is reduced, the results suggest that composite prevalence estimates are robust to decline of assay sensitivity. As shown in Table 1a, when sensitivity is decreased from 0.95, to 0.90, 0.85, 0.80, composite prevalence estimates do not change much and the estimated-to-true ratios remain similar. When genotyping assay sensitivity is held unchanged and specificity is decreased from 0.95, to 0.90, 0.85, 0.80, composite prevalence estimates increase and the estimated-to-true ratios become much larger (Table 1b). The overestimating problem is alleviated when the number of HPV types in the composite measure becomes larger.

Simulation results also suggest that the composite prevalence estimates could under-estimate vaccine effectiveness (Table 1a,b). When the prespecified “true” underlying type-specific prevalence of HPV 6, 11, 16 and 18 are reduced to 50% of the reported level and genotyping assay with sensitivity and specificity (0.95.0.95) is used, the prevalence estimates of 2 high-risk vaccine types and 4 vaccine types were only reduced by 17.2% and 16.7%, respectively, which are much lower than the 50% and 48% reduction in the prespecified infection burden.

Table 2(a,b) shows the results of estimated composite prevalence when “true” type-specific prevalence is prespecified as the reported prevalence in the Northwest Territories (NWT), Canada. Results suggest that the magnitude of over-estimation is greater in the NWT, Canada than in the U.S. due to lower HPV infection burdens in Canada. In addition, increasing the number of HPV types in the composite measure does not help much to alleviate the over-estimating problem as in the U.S. scenarios.

**Table 2:** **Composite prevalence estimates when the “true” underlying type-specific infection burden is pre-specified as the reported prevalence in the Northwest Territories, Canada**

No. of HPV types*	**Time** ^**§**^	**Pre-specified true composite prevalence (SD)**	**Composite prevalence estimate (SD)**	**Est. to true ratio**	**Composite prevalence estimate (SD)**	**Est. to true ratio**	**Composite prevalence estimate (SD)**	**Est. to true ratio**	**Composite prevalence estimate (SD)**	**Est. to true ratio**
**a: Sensitivity ≤ Specificity**										
			**Sen. = 0.95**		**Sen. = 0.90**		**Sen. = 0.85**		**Sen. = 0.80**	
			**Spe. = 0.95**		**Spe. = 0.95**		**Spe. = 0.95**		**Spe. = 0.95**	
2	Baseline	0.053 (0.004)	0.142 (0.006)	2.676	0.140 (0.005)	2.642	0.138 (0.005)	2.604	0.135 (0.005)	2.547
	Reduced	0.027 (0.003)	0.120 (0.005)	4.444	0.119 (0.005)	4.407	0.117 (0.005)	4.333	0.116 (0.005)	4.296
	% red.	49.1	15.5		15.0		15.2		14.1	
4	Baseline	0.064 (0.004)	0.231 (0.007)	3.609	0.229 (0.006)	3.578	0.226 (0.006)	3.531	0.224 (0.006)	3.500
	Reduced	0.033 (0.003)	0.207 (0.006)	6.272	0.206 (0.006)	6.242	0.204 (0.006)	6.181	0.203 (0.007)	6.152
	% red.	48.4	10.4		10.0		9.7		9.4	
22	Baseline	0.216 (0.006)	0.701 (0.007)	3.245	0.697 (0.007)	3.227	0.694 (0.004)	3.213	0.690 (0.007)	3.194
	Reduced	0.194 (0.006)	0.694 (0.007)	3.577	0.691 (0.007)	3.562	0.687 (0.007)	3.541	0.684 (0.007)	3.526
	% red.	10.2	0.9		0.9		1.0		0.9	
45	Baseline	0.287 (0.007)	0.841 (0.006)	2.930	0.839 (0.006)	2.923	0.836 (0.006)	2.913	0.834 (0.006)	2.906
	Reduced	0.265 (0.007)	0.838 (0.006)	3.162	0.835 (0.006)	3.151	0.833 (0.006)	3.143	0.831 (0.006)	3.136
	% red.	7.6	0.4		0.5		0.4		0.4	
**b: Sensitivity ≥ Specificity**										
			**Sen. = 0.95**		**Sen. = 0.95**		**Sen. = 0.95**		**Sen. = 0.95**	
			**Spe. = 0.95**		**Spe. = 0.90**		**Spe. = 0.85**		**Spe. = 0.80**	
2	Baseline	0.052 (0.004)	0.142 (0.006)	2.731	0.229 (0.006)	4.404	0.311 (0.007)	5.981	0.387 (0.008)	7.442
	Reduced	0.027 (0.003)	0.120 (0.005)	4.444	0.209 (0.007)	7.74	0.293 (0.007)	10.85	0.371 (0.008)	13.74
	% red.	49.1	15.5		8.7		5.8		4.1	
4	Baseline	0.064 (0.004)	0.231 (0.007)	3.609	0.375 (0.007)	5.859	0.497 (0.008)	7.766	0.599 (0.008)	9.359
	Reduced	0.033 (0.003)	0.207 (0.006)	6.272	0.356 (0.008)	10.78	0.482 (0.009)	14.61	0.588 (0.008)	17.82
	% red.	48.4	10.4		5.1		3.0		1.8	
22	Baseline	0.216 (0.007)	0.701 (0.007)	3.245	0.883 (0.005)	4.088	0.954 (0.003)	4.417	0.983 (0.002)	4.551
	Reduced	0.194 (0.006)	0.694 (0.007)	3.577	0.881 (0.005)	4.541	0.954 (0.003)	4.917	0.982 (0.002)	5.062
	% red.	10.2	0.9		0.2		0		0.1	
45	Baseline	0.288 (0.007)	0.841 (0.006)	2.920	0.961 (0.003)	3.337	0.990 (0.002)	3.438	0.997 (0.001)	3.462
	Reduced	0.265 (0.007)	0.838 (0.006)	3.162	0.960 (0.003)	3.623	0.990 (0.002)	3.736	0.997 (0.001)	3.762
	% red.	7.6	0.4		0.1		0		0	

*2: high-risk vaccine types(HPV 16, 18); 4: vaccine types (HPV 6, 11, 16, 18); 23: high-risk types (HPV 16, 18, 26, 30, 31, 33, 35, 39, 45, 51, 52, 53, 56, 58, 59, 66, 67, 68, 69, 70, 73, 82, 85); 45: any types (HPV 6, 11, 13, 16, 18, 26, 30, 31, 32, 33, 35, 39, 40, 42, 43, 44, 45, 51, 52, 53, 54, 56, 58, 59, 61, 62, 66, 67, 68, 69, 70, 71, 72, 73, 74, 81, 82, 83, 84, 85, 86, 87, 89, 90, 91).

^§^Pre-specified type-specific prevalence at baseline:HPV6 = 0.009, HPV11 = 0.003, HPV13 = 0.001, HPV16 = 0.041, HPV18 = 0.013, HPV26 = 0, HPV30 = 0.002, HPV31 = 0.021, HPV32 = 0.001, HPV33 = 0.007, HPV35 = 0.007, HPV39 = 0.016, HPV40 = 0.005, HPV42 = 0.013, HPV43 = 0.002, HPV44 = 0.003, HPV45 = 0.015, HPV51 = 0.016, HPV52 = 0.014, HPV53 = 0.005,HPV54 = 0.008, HPV56 = 0.010, HPV58 = 0.013, HPV59 = 0.012, HPV62 = 0.016, HPV66 = 0.019, HPV67 = 0.013, HPV68 = 0.003, HPV69 = 0.003, HPV70 = 0.011, HPV72 = 0.007, HPV73 = 0.005, HPV74 = 0.007, HPV81 = 0.007, HPV82 = 0.002, HPV83 = 0.007, HPV84 = 0.004, HPV85 = 0.003, HPV89 = 0.010, HPV90 = 0.004, HPV86 = 0.004, HPV87 = 0.001, HPV91 = 0.001, HPV61 = 0.001, HPV71 = 0.

Reduced: vaccine types (HPV 6, 11, 16, 18) are reduced 50%.

The results of sensitivity analysis for correlations are given in Table 3. Results suggest that estimated-to-true prevalence ratios are robust to the change of correlations and the impact of correlations on the composite measures depends on the magnitude of correlations and the number of HPV types in the composite measures. The impact of correlations is limited when the number of HPV types in the composite measure is relatively small (e.g., 2, 4). Magnitude of decline in composite prevalence estimates increases with increasing number of HPV types in the composite measures (Table 3).

**Table 3:** **Sensitivity analysis for correlations**

	2 High-risk vaccine type*			4 Vaccine type*			14 High-risk type*			37 HPV type*		
**Correlations**	**Prespecified true Composite prevalence**	**Composite prevalence estimate** ^**§**^	**Est. to true ratio**	**Prespecifiedtrue composite prevalence**	**Composite prevalence estimate** ^**§**^	**Est. to true ratio**	**Prespecified true composite prevalence**	**Composite prevalence estimate** ^**§**^	**Est. to true ratio**	**Prespecified true composite prevalence**	**Composite prevalence estimate** ^**§**^	**Est. to true ratio**
0	0.064	0.151	2.36	0.121	0.279	2.30	0.293	0.647	2.23	0.663	0.947	1.43
0.05	0.064	0.151	2.36	0.121	0.275	2.27	0.282	0.616	2.19	0.617	0.904	1.47
0.1	0.064	0.151	2.36	0.119	0.269	2.26	0.271	0.584	2.15	0.576	0.861	1.49
0.2	0.063	0.147	2.33	0.115	0.255	2.22	0.247	0.526	2.13	0.501	0.776	1.55
0.3	0.062	0.144	2.32	0.110	0.242	2.20	0.223	0.473	2.12	0.436	0.695	1.59
0.4	0.061	0.141	2.31	0.104	0.228	2.19	0.200	0.423	2.12	0.376	0.615	1.63

*2: high-risk vaccine types (HPV 16, 18); 4: vaccine types (HPV 6, 11, 16, 18); 14: high-risk types (HPV 16, 18, 31, 33, 35, 39, 45, 51, 52, 56, 58, 59, 66, 68); 37: HPV types (HPV 6, 11, 16, 18, 26, 31, 33, 35, 39, 40, 42, 45, 51, 52, 53, 54, 55, 56, 58, 59,61, 62, 64, 66, 67, 68, 69, 70, 71, 72, 73, 81, 82, 83, 84, 89, IS39).

Pre-specified type-specific prevalence: HPV 6 = 0.028, HPV11 = 0.034, HPV16 = 0.047, HPV18 = 0.019, HPV26 = 0.022, HPV31 = 0.015, HPV33 = 0.013, HPV35 = 0.023, HPV39 = 0.011, HPV40 = 0.018, HPV42 = 0.025, HPV45 = 0.020, HPV51 = 0.041, HPV52 = 0.036, HPV53 = 0.058, HPV54 = 0.038, HPV55 = 0.024, HPV56 = 0.023, HPV58 = 0.014, HPV59 = 0.030, HPV61 = 0.040, HPV62 = 0.065, HPV64 = 0.002, HPV66 = 0.034, HPV67 = 0.013, HPV68 = 0.016, HPV69 = 0.027, HPV70 = 0.020, HPV71 = 0.056, HPV72 = 0.012, HPV73 = 0.015, HPV81 = 0.027, HPV82 = 0.054, HPV83 = 0.041, HPV84 = 0.048, HPV89 = 0.047, HPVIS39 = 0.014.

Pre-specified genotyping assay sensitivity = 0.95 and specificity = 0.95.

^§^Standard deviations of prevalence estimates ≤0.008.

## Discussion

Prevalence estimates based on one or panels of PCR genotyping assay results have been used to assess HPV infection burdens and monitor vaccine effectiveness [11,29]. Since true HPV infection burdens are often unknown, mathematical algorithms with prespecified infection burdens and assay performance were used to simulate various scenarios to evaluate these composite prevalence estimates.

PCR-based DNA genotyping tests can detect the existence of small amount of virus. The PCR process includes denaturation, annealing and extension in each PCR cycle. Each cycle approximately doubles the amount of target viral DNA. Although PCR process is labor-intensive and time consuming, PCR can theoretically produce one million copies from a single double-stranded DNA molecule after 30 cycles. Analysis of the amplification products can be done in different ways including gel electrophoresis, dot blot or line strip hybridization [30].

The initial values of PCR genotyping assay sensitivity and specificity were set to be equal to (0.95, 0.95) in this simulation study to reflect a well-performed Linear Array test. Roche Linear Array genotyping assay is the commercialized version of a PCR test which is designed to standardize the entire PCR process and to detect 37 HPV types. LA is a qualitative test and has been used for research purpose in numerous epidemiological/clinical studies. It is also the most widely-used assay by the labs of the World Health Organization (WHO) HPV labNet to monitor HPV vaccine effectiveness. When specimens are carefully handled and PCR procedures are strictly performed according to protocol, analytical sensitivity and specificity of Linear Array assay can reach (0.95, 0.95). The performance of in-house assays can have greater variation since each of the many steps of the PCR testing procedure can introduce important variability [18-20]. Factors associated with analytical sensitivity and specificity of PCR testing include primer selection, lab environment and reaction conditions, performance of the DNA polymerase used in the reaction, laboratory techniques of personnel and specimen acquisition, handling and storage, etc. [30].

Type-specific HPV infections are considered to be correlated in this simulation study because the risk factors of getting infected by various HPV types are similar and subjects with weaker immune systems are more likely to get infected and/or stay infected. Without loss of generality, the correlations between HPV types were set to be 0.05. This is the mean of 666 pairwise correlations calculated based on 37 Linear Array genotyping test results collected in the 2003–2006 National Health and Nutrition Examination Survey (NHANES). Although the values of pairwise correlations varied from 0 to 0.3, eighty-five percent of these pairwise correlations were somewhere between 0 and 0.1. In addition, the simulation results (Table 3) suggest the effect of correlations on estimated-to-true prevalence ratios is limited. The impact of correlations on the composite prevalence depends on the magnitude of correlations and the number of HPV types in the composite measure. The higher the correlations between different HPV types, the more likely co-infections would occur. Therefore, the higher the co-infection rate, the lower the composite prevalence will be.

When comparing results from the U.S. scenarios with those from the NWT, Canada, although results are generally consistent, the magnitude of over-estimation is more severe in the NWT, Canada scenarios. This is because the pre-specified “true” underlying type-specific infection burden is generally lower in the NWT, Canada and the chance of getting false positive increases. Assay specificity become even more important for getting accurate prevalence estimates. Unlike the US scenario, increasing number of HPV types in the composite measures does not always help to ease the overestimating problem in the NWT, Canada. It is because in the U.S. scenarios, the type-specific infection burdens of newly-added HPV types are at similar or higher levels than those already in the composite measure. In the NWT, Canada, the type-specific infection burdens of newly-added types are much lower than those already in the composite measure (Figure 1), therefore, the magnitude of false positive rates increased is much greater.

In the context of monitoring HPV infection burden, the focus has been given to assay analytical sensitivity to detect the existence of HPV infections. There has been a tendency either to develop a new testing technique or to modify existing techniques to increase analytical sensitivity to detect HPV. Studies suggest that increasing analytical sensitivities of HPV detection has reveals that the HPV prevalence is higher than previously suggested [31,32]. In contrast to previous studies, the simulation results suggest that prevalence estimates based on PCR genotyping assay results generally overestimate the true infection burden; genotyping assay sensitivity has limited effect on the composite prevalence estimates; and the decline in specificity is more influential. When assay specificity declines, false positive rates increase and the problem of overestimating becomes more severe. Particularly, when underlying type-specific infection rates are low, for each HPV type, small reductions in genotyping assay specificity results in a high number of subjects with false positive results. Therefore, eliminating factors which might cause false positives (i.e., contamination introduced through reagents, laboratory disposables or equipment including carry-over contamination between tests or sample-to-sample contamination, etc.) to increase assay specificity is important.

Since the introduction of the HPV vaccine in 2006, more nations are now monitoring HPV infection as an earlier indication of vaccine effectiveness [11,12,29]. More than 20 types of in-house or commercial assays have been developed to detect HPV infections and the performance of these assays varies [13,16,18,20]. Also, true underlying infection burdens vary by geographic regions, age groups [16,17] and can change over time. We need to be aware that accuracy of prevalence estimates based on panels of genotyping assay results can vary by true underlying infection burden, genotyping assay performance and number of HPV types included in the composite measure. For geographic regions or subpopulations with relatively low infection burden, in general, prevalence estimates overestimate true underlying infection burden and could underestimate vaccine effectiveness. Also, we need to understand that the impact of genotyping assay specificity is as or more important than sensitivity and should be considered in selecting a genotyping assay to monitor HPV infections. Particularly, subjects with positive HPV detected by PCR in research studies generally are not informed or referred to have colposcopy, since persistent infection of HPV high-risk types is the pivotal event in the development of cervical cancer and most of HPV detected can be cleared without treatment in about two years. In addition, on the population level, from the point of view of infectiousness, population prevalence calculations based on PCR results could overestimate true infectiousness burden, since a very tiny amount of DNA detected by PCR is likely not infectious and may just represent a past infection. Furthermore, laboratory guidelines or policies leading to more standardized assay performance between different laboratories are necessary for combining data from different sites to estimate vaccine effectiveness or compare infection burden at various geographic regions.

In this study, we simulated various scenarios to evaluate composite prevalence estimates based on PCR genotyping assay results. Although it is not possible to consider all levels of infection burden or PCR genotyping assay performance, this simulation study is able to examine the impact of true infection burden and assay sensitivity and specificity on the accuracy of composite prevalence estimates. Estimated-to-true prevalence ratios were used to examine how well the prevalence estimates based on genotyping assay results measure the true underlying infection burden. One limitation is the ratio does not provide information to distinguish true and false positive rates. True and false positive rates depend on the type-specific infection burden and genotyping assay sensitivity and specificity. Although the simulation result suggest that increasing number of HPV types in the composite measure could improve the accuracy of composite prevalence, HPV types are grouped to form composite measures based on their association with a variety of clinical conditions, phylogenetic position or types related to vaccines and may not be varied. Cross-reaction is not discussed in this manuscript since the chance of cross-reaction occurrence when applying PCR testing technique is relatively low. In addition, bias which can be introduced due to study design (i.e., sampling strategy, confounders) is not discussed in this manuscript. Having a good sample representing the target population is very important. Since PCR genotyping assay results have limited clinical utility, future studies can be conducted to investigate incorporating HPV clinical tests (i.e.,Digene HC2 or Cobas test) to monitor HPV infections.

## Conclusions

Composite prevalence estimates calculated based on panels of genotyping assay results generally overestimate the true infection burden and could underestimate effectiveness. Analytical specificity of genotyping assay is as or more important than sensitivity and should be considered in selecting assay to monitor HPV.

## Appendix

Suppose that n independent subjects, i = 1,2,…,n, are in the study. For each subject, i, K various HPV types, j = 1,2,….k, are tested. Let δ_j_ denote the true type-specific prevalence for the j-th HPV type and δ_j_s = (δ_1_, δ_2_,…,δ_j_,…,δ_k_) be the true type-specific prevalence for k various types. The true type-specific prevalence rates are considered to be different. α and β are the analytical sensitivity and specificity of genotyping assays. Genotying assay performance is considered to be the same for all types. Let T_i1_, T_i2_,..T_ij_,…,T_ik_ be the genotyping assay results of k various types of the i-th subject and T_ij_ be the j-th type-specific result of the i-th subject. Let D_i1_, D_i2_,…,D_ij_,…, D_ik_ be the true infection status of k various types of the i-th subject.

Similar to the latent approach in Lin et al. for subunit diagnostic tests [26], we assume that true type-specific infection status and genotyping test result of k various types, (D_i1_,T_i1_, … D_ij,_ T_ij_, …, D_ik_,T_ik_), is a manifestation of a multivariate normal latent vector (Y_i1_, Z_i1_,…Y_ij_, Z_ij_,…, Y_ik_, Z_ik_) with mean zero and correlation matrix ∑_k_, a 2 k by 2 k matrix. Without loss of generality, the variance of Y_ij_ and Z_ij_ are assumed to be one. For each type, j, Y_ij_ and Z_ij_ are latent variables with thresholds of p_j_ and q_j_ for the binary variables, D_ij_ and T_ij_, respectively. In another words, Y_ij_ and Z_ij_ are dichotomized as follows:

$$ {D}_{ij}=\left\{\begin{array}{l}1\kern0.36em  if\kern0.24em {Y}_{ij}\ge {p}_j\hfill \\ {}0\kern0.24em  if\kern0.24em {Y}_{ij}<{p}_j,\hfill \end{array}\right. $$

$$ {T}_{ij}=\left\{\begin{array}{l}1\kern0.36em  if\kern0.24em {Z}_{ij}\ge {q}_j\hfill \\ {}0\kern0.24em  if\kern0.24em {Z}_{ij}<{q}_j.\hfill \end{array}\right. $$

The threshold values of p_j_ can be expressed as functions of the true infection rate of the j-th type, δ_j_, and the threshold values of q_j_ can be expressed as functions of the true type-specific prevalence of the j-th type, δ_j_, assay sensitivity, α, and specificity, β, as follows:

$$ {p}_j={\varPhi}^{-1}\left({\delta}_j\right)\;\boldsymbol{and}\;{q}_j={\varPhi}^{-1}\left(\alpha {\delta}_j+\left(1-\upbeta \right)\left(1-{\delta}_j\right)\right), $$

where ΦΟ is the standard cumulative univariate normal distribution function. It is because

$$ P\left({Y}_{ij}\ge {p}_j\right)=P\left({D}_{ij}=1\right)={\delta}_j, $$

and

$$ \begin{array}{l}P\left({Z}_{ij}\ge {q}_j\right)=P\left({T}_{ij}=1\right)\hfill \\ {}\kern3em =P\left({T}_{ij}=1,{D}_{ij}=1\right)+P\left({T}_{ij}=1,{D}_{ij}=0\right)\hfill \\ {}\kern3em =P\left({T}_{ij}=1\left|{D}_{ij}=1\right.\right)P\left({D}_{ij}=1\right)+P\left({T}_{ij}=1\left|{D}_{ij}=0\right.\right)P\left({D}_{ij}=0\right)\hfill \\ {}\kern3em =\alpha {\delta}_j+\left(1-\beta \right)\left(1-{\delta}_j\right).\hfill \end{array} $$

Furthermore, α and p_j_, and q_j_ (and thus α, β, and δ_j_) also determine the correlation between Y_ij_ and Z_ij_, since

$$ \alpha =P\left({T}_{ij}=1\left|{D}_{ij}=1\right.\right)=\frac{P\left({Y}_{ij}\ge {p}_j,{Z}_{ij}\ge {q}_j\right)}{P\left({Y}_{ij}\ge {p}_j\right)}=\frac{1-\Phi \left({p}_j\right)-\Phi \left({q}_j\right)+{\Phi}_2\left({p}_j,{q}_j;\upvarrho \right)}{1-\Phi \left({p}_j\right)} $$

where φ_2_ (y,z;ϱ) is the cumulative distribution function of bivariate normal random vector with means of zero, variance of 1 and correlation of ϱ. A numerical method is used to solve for ϱ with a given α, β and δ_j_.

To generate the data, for each type, we first identify the threshold values of p_j_ and q_j_ based the pre-specified values of δ_j_, α, β. Second, we identify correlation matrix ∑_k_ (where correlation between each pair of Y_j_ and Z_j_ is obtained by a numerical method). Multivariate normal data (Y_i1_, Z_i1_,…Y_ij_, Z_ij_,….,Y_ik_, Z_ik_) are generated with mean zero and correlation matrix ∑_k_. Then, for each HPV type, we use the pre-determined threshold values of p_j_ and q_j_ to dichotomize Y_ij_ and Z_ij_ to obtain D_ij_ and T_ij_.

To illustrate, when α = 0.95, β = 0.95 and δ_j_s = (0.0283, 0.0339, 0.0469, 0.0185),

$$ {\displaystyle {\sum}_4=\left(\begin{array}{llllllll}1\hfill & 0.91\hfill & 0.05\hfill & 0.05\hfill & 0.05\hfill & 0.05\hfill & 0.05\hfill & 0.05\hfill \\ {}\hfill & 1\hfill & 0.05\hfill & 0.05\hfill & 0.05\hfill & 0.05\hfill & 0.05\hfill & 0.05\hfill \\ {}\hfill & \hfill & 1\hfill & 0.92\hfill & 0.05\hfill & 0.05\hfill & 0.05\hfill & 0.05\hfill \\ {}\hfill & \hfill & \hfill & 1\hfill & 0.05\hfill & 0.05\hfill & 0.05\hfill & 0.05\hfill \\ {}\hfill & \hfill & \hfill & \hfill & 1\hfill & 0.93\hfill & 0.05\hfill & 0.05\hfill \\ {}\hfill & \hfill & \hfill & \hfill & \hfill & 1\hfill & 0.05\hfill & 0.05\hfill \\ {}\hfill & \hfill & \hfill & \hfill & \hfill & \hfill & 1\hfill & 0.89\hfill \\ {}\hfill & \hfill & \hfill & \hfill & \hfill & \hfill & \hfill & 1\hfill \end{array}\right)} $$ is used to generate the laten vectors.
